# The use of PLANS and NetworkX in modeling power grid system failures

**DOI:** 10.1038/s41598-022-22268-z

**Published:** 2022-10-19

**Authors:** Piotr Hadaj, Dominik Strzałka, Marek Nowak, Małgorzata Łatka, Paweł Dymora

**Affiliations:** Faculty of Electrical and Computer Engineering, Rzeszów University of Technology, Al. Powstańców Warszawy 12, 35-959 Rzeszów, Poland

**Keywords:** Energy science and technology, Electrical and electronic engineering

## Abstract

The theoretical and practical aspects and results of simulations based on a specialized tool that is used in the energy industry were adressed. The previously discussed cases in the literature by taking into account the worst case and critical states of networks in terms of complex networks were extended. Using the Monte-Carlo method, the vulnerability of the power grid to node failures was investigated, both in terms of the use of specialized software, which is used in the power industry, and a tool for the analysis of complex networks graphs. We present the results obtained and the observed analogy between the results of the analysis performed in specialized software and the complex network graph analysis tool. It has been shown that the results obtained coincide for both software packages, even though their application focuses on slightly different aspects of system operation. Moreover, further possibilities of extending the research in this direction are proposed, taking into account not only the improvement of the method used, but also a significant increase in the size of the tested structure model.

## Introduction

The nowadays progress in power engineering modeling and simulation is supported by computer packages that allow finding stable and critical system states specified by interconnected power system (IPS) setups combining many individual operating electric power systems (EPS). The main goal of such activity is the improvement of power supply reliability^1^, to provide high electric energy quality for consumers^2^, to find more efficient use of available power-generating system capacity^3^. The increasing number of such grids elements (transmission lines, transformers, generators, nodes, etc.) suggests that IPS’s with high-voltage transmission main and back bones should be considered in terms of complex networks^4–6^. In such networks we can observe a high possibility of emergency states due to operational breakdowns. The issue of transmission network resilience has also been studied more extensively by other researchers in classical approach, such as^7^ where the authors focus on tips and methods for getting a collapsed system back up and running quickly, or also from the aspect of complex network theory, such as^8^ or^9^. One can check each random IPS state using reliability analysis (RA) and test the electrical operation mode tolerability using Ohm’s and Kirchhoff’s laws. This allows transmission system operators (TSO) and distribution system operators (DSO) providing uninterrupted, sustainable and reliable delivery of electrical power to consumers located in a relatively small area. However, the hand checking process can last for a relatively long time and does not allow testing further possible scenarios including prediction of power grid behavior in short-term future.

It is obvious that the increasing complexity of analyzed power systems and expectations related to short- and long-term stability predictions require the support of advanced computer technologies. One can observe a paradigm shift from individual manual made calculations with the use of simple software to more complex software solutions designed for operation analysis tasks dispatch management decision-making. The automation of calculations allows solving many important problems related to RA because the maximum number of significant element outages in IPS’s is not always known, thus the focus on systems reliability analysis only having the utmost combination of dead elements is infeasible^3^. A large sequence of calculations to determine RA of IPS cannot be implemented without the support of advanced computer technology and among known solutions we can indicate:

- PLANS^10^,
- GridLAB-D^11^,
- PowerWorld^12^,
- SYNDIS^13^,
- GE Positive Sequence Load Flow Software (GE-PSLF)^14^,
- NEPLAN^15^.

**Figure 1:**
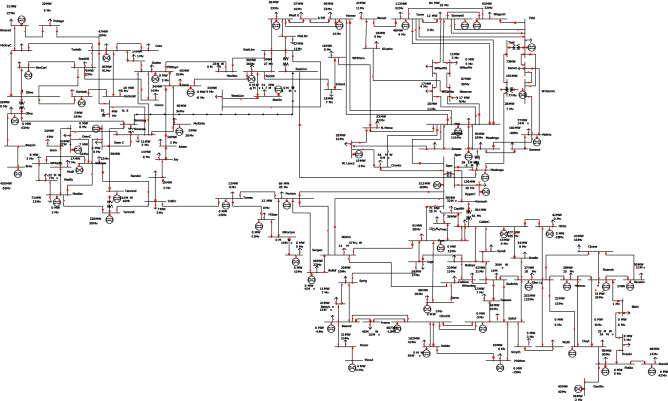
Original power grid model (PowerWorld visualization).

Apart on the problems related to IT tools supporting power engineering modeling and simulation another problem is the lack of real power grid networks data. Having in mind that for each country power grid system is important element of its critical infrastructure, many specific and detailed data aren’t and cannot be available. Thus for calculation experiments we are forced to use some existing, historical data sets. The original IEEE118-Bus System was derived from a portion of the American Electric Power System (in the Midwestern US) as of December, 1962 (see Fig. 1). This scheme was reconfigured for the three western regions of the United States. It is a basic power systems test case that was considered in many papers during last 60 years^16–19^.

The IEEE118-Bus Test Case data was entered to computer systems manually and ”*made available to the electric utility industry as a standard test case*”^3^. It was primarily in PECO PSAP format (entered by Rich Christie, researcher at University of Washington in 1993)^20^. Later he converted it into IEEE Common Data Format. Most of the bus names in the original model, were read from line diagram distributed with the package. The names were hardly readable, printed in a small font, and after many generations of copying, errors of transcription may have occurred. The unreadable and missing bus names were simply made up. Voltage levels in the bus names seem to be a bad guess. Also the line MVA limits were made up. As this was widely used test case, it had a lot of voltage control devices, nodes, and is quite robust in detecting network and software vulnerabilities using simulations in various cases.

There is also new version of this simulation model, named NREL-118. The new database is based on old model, but it represents three regions of the United States Western Interconnection. Model includes over one year of time-synchronous data, including wind, solar time series and hourly load of the system^18^. There are also different test models, such as^21^, which incorporates equivalent 100 MW wind power plant (WPP) and its associated unit transformer, collector system, and substation transformer.

Attempts already have been made to analyze not only the original but also new model^22^, but so far this has been done on the original form of the data, based on the US transmission system. In our case, the model was adopted and reconfigured to the part of Polish energy system conditions for middle summer and winter. One of the important paper’s contributions is the conversion of model data into KDM and mathematical graph formats (details are given in “Case study” section). Additionally, some names, properties and parameters of nodes and transmission branches between them were modified according to real, but probably in some cases approximated data related to the parameters of Polish power grid. It is worth mentioning at this point that a few years ago, there used to be a difference between summer and winter time, and now because the air conditioning systems are commonly used, the differences are basically negligible^23^.

The paper is organized as follows. “Complex networks and power grids” section describes complex networks theory and parameters. “PLANS” and “Calculation method” sections is about PLANS and calculation method used in it. The Monte Carlo method, on which the algorithm for performing simulations is based, is taken up in the “Monte Carlo” section. Part “Case study” section describes a case study of the performed simulation and the obtained results, both for PLANS and NetworkX. Finally, “Summary” section presents conclusions and proposed methods for developing further research.

## Complex networks and power grids

In 1959, Hungarian mathematicians Paul Erdös and Alfréd Rényi proposed the random graph model^24^. They assumed that connections (edges) in a real network (graph) are formed randomly, and therefore the probability calculus can be used to analyses them. This was the dominant model of analysis until the development of computers with sufficient computing power, which made it possible to analyses networks with millions of nodes. It turned out for instance that the distribution of the number of connections to each node in many (complex) networks is governed by power-law distributions, not by normal ones, as would result from the random graph model. This discovery is attributed to Albert László Barabási. Thanks to him, new questions began to be asked leading to an understanding of the network as a topology of interactions between its elements. Many natural and social systems are in fact complex networks, and although traditionally these systems have been modeled using random graph theory, it is increasingly recognized that their topology and evolutionary process are not random^25^. Usually it is considered that complex networks are examples of complex systems^26^.

In terms of mathematical definition, the idea of (complex) system *S* can be seen as a being *B* represented by *n*-th element set of topology nodes $E = \{e_1, e_2, \ldots e_n\}$. These elements have *m* attributes (the set $A = \{a_1, a_2, \ldots , a_m\}$) and there are *k* possible long- or short-range relations between them (edges in graph topology) given by the set $R = \{r_1, r_2, \ldots , r_k\}$. Finally, we have $S = B(E, A, R)$^27^. There is no one commonly accepted definition of complex systems^28–30^ but most of existing definitions refer the Aristotle’s rule: the whole is more than the sum of its parts^31^. In the case of electrical power grid systems we can refer *E* to set of nodes (power stations, transformers, loads and generators), *A* to nodes attributes such as: active power, reactive power, node voltage modulus, phase angle of the voltage. *R* refers to power lines between these nodes.

In literature we can find many approaches to model power grids as complex networks, starting from^32^, where the classical definition of complex networks and an approach to multiple structures modeling as complex networks, not just electrical networks, is given. In paper^4^ there is a proposal to apply the theory of complex networks to investigate the behavior of interconnected electrical and IT networks. Results show that the coupled infrastructure complies with the typical characteristics of scale-free network and among all topological indices, efficiency and EWND (Efficiency-weighted Node Degree) analysis are most effective in identifying critical interconnected components.

In a very recent work^33^, authors consider power grids as cyber-physical systems, that can be modeled as network systems. The review is accompanied by some simulations on benchmark and real power grids to show the applicability of these concepts. Another paper^34^ presents the main characteristics of power grids, together with the possibilities of making them into smart grids. In addition, there is a study of the impact of electric line overloads on the nature of blackouts.

In most cases these approaches are based on topological properties analyses of connection networks but if such analyses are not related to simulations based on the software utilized for loads and flows calculations in power grids the final results are not reliable and are not convincing especially for DSO’s^6^. Simulations performed in specialized software undoubtedly give a more complete insight into the behavior of the network, as they are able to reflect most of the characteristic parameters that cannot be represented in a simple graph of the complex network^2^. There is also another approach to the simulation problem^35^, where the authors focused on using a modified Hirsch index method, testing its performance on a different test set (IEEE-39 bus).

Graph *G* can be interpreted as weighted (between each node *i* and *j* there is a value $\ell _{ij}$) or also unweighted. The second case means, that only the existence of connection is considered, but for weighted graph edge weights are required $\ell _{ij}$, which may represent physical distance, transmission cost, transmission time, transmission speed, or link capacity. An unweighted graph is a special case of a weighted graph, where all the edge weights $\ell _{ij}$ have value 1, $\forall i\ne j$. For every two nodes of the graph, the shortest path length $d_{ij}$ can be determined, as the smallest sum of the weights $\ell _{ij}$ of all possible paths between vertices *i* and *j*, $d_{ij}\ge \ell _{ij}$, $\forall i \ne j$. It is also possible to define the efficiency of the edge (path) as $\varepsilon _{ij} = 1/ d_{ij}$, $\forall i \ne j$. If $d_{ij} =\infty$, then $\varepsilon _{ij} = 0$. The shortest path between *i* and *j* can be interpreted as high efficiency, and in the range of the whole graph it can be defined as^36^:

1 $$\begin{aligned} E(G)=\frac{1}{N(N-1)}\sum _{i\ne j\in G}\frac{1}{d_{ij}}. \end{aligned}$$

In Eq. (1), where $d_{ij}$ defines the shortest possible path between *i* and *j* nodes (vertices), it is relatively easy to calculate the average efficiency of the whole network. There are two types of efficiency parameters: general and local. General [defined by Eq. (1)] refers to the whole network, and for each vertex *i* of the graph there is a possibility to define the local efficiency $E_{loc}(G_{i})$, which is the average efficiency of the local subgraphs, and can be interpreted as the general fault tolerance of the system; it also indicates the efficient communication between the first neighbors of node *i*, when *i* is removed^36^. This can be in some cases interpreted as the cost of operating the entire network, including the cost of energy transmission. If the efficiency of the network decreases, the costs increase (e.g. in information transport).

The existence of connections between the nearest neighbors of the vertex *i* can be defined by the *C* coefficient. Lets consider, that vertex *i* of a graph has $k_i$ connections (edges) with other vertices $k_i$. All of the vertices will be neighbors of the vertex *i*^37^:

2 $$\begin{aligned} \left( \begin{matrix}k_i\\ 2\\ \end{matrix}\right) =\frac{k_i(k_i-1)}{2}. \end{aligned}$$

Considering the above, the clustering coefficient $C_{i}$ for vertex *i* can be defined as a ratio between the number of edges $E_{i}$ that connects these vertices $k_{i}$ and total possible number of node connections (3). Average value of $C_{i}$ for all *i* is referred as the clustering coefficient for whole graph:

3 $$\begin{aligned} C_i=\frac{2E_i}{k_i(k_i-1)}. \end{aligned}$$

In the case of network analysis, different parameters can be analyzed. In this case, global and local efficiency were taken into account. The authors believe that the selected parameters adequately describe the state of the network structure when the worst scenario is assumed in both simulation softwares, namely PLANS and NetworkX.

It is not easy to understand the vulnerability nature of networks. In paper^38^, authors provide a detailed analysis of the impact of link failures in linear flow networks and focus, among others, on the network response and spatial flow rerouting. It turns out that is possible to forecast flow rerouting after network failures based on purely topological measures. In another article^39^, authors have shown a general complex network failure analyzing framework and, what is even more interesting, the proof to the existence of network isolators (certain subgraphs) that are able to suppress the occurrence of any failure spreading, and show how to create such structures both in synthetic and real-world networks.

In papers^4^ and^35^ the problem of network efficiency is analyzed from the topological point of view, taking into account only test bus systems, which do not necessarily reflect the current operating conditions and network tasks. The real challenge in power grid network is to deliver the demanded amount of electrical energy to different nodes with (physical) limitations related to the edges and nodes in topological structure. The number of practical examples dealing with real input data related to power grid topology and processed task is quite low. So far many authors focused on topological aspects of networks properties, parameters, their stability, possible dynamics of phenomenon, etc., however, in order to find a global spatio-temporal description of (complex) systems that helps a better understanding of their real nature the holistic, interdisciplinary approach can be used. It is important to focus on system (network) topology, its (sub)parts, how they are connected together and also what kind of tasks are processed in the system (network). An example can be a computer network where the topological network properties (topology) can be analyzed and the nature of network traffic (processed task). The first one refers to spatial domain whereas the second one to time domain. In contradiction to computer networks, where after the failure of some network nodes there are some built-in mechanism allowing to reroute (reconfigure the network topology) the information packets, in power grid network the existing topology is used to transfer electrical energy and the network reconfiguration (if possible) may not be enough to ensure the network stability. Network edges and nodes are fused in order to avoid the overload—the flow of current is higher as it is physically possible due to the node and connection (electrical) parameters, whereas in computer network this could cause the longer router queue that finally leads to waiting time increase.

The investigations related to simulations of theoretical results are interesting but from engineering point of view it is also valuable how some issues can be solved practically. In this paper, the main focus is on the application of computer simulation software. PLANS is a software that is used by at least 4 of 5 Polish (but not only, it is used for academic activity including teaching and research) DSO operators for modeling and simulations of different states of Polish power grid system. The authors of this software successfully used it for many years and prepared many important simulations for working, reconfigured, re-built and reorganized parts of Poland’s power grid network. According to PLANS user manual^10^, anytime when during the simulations the non-convergence message is visible such situation is considered as system failure.

PLANS is based on mathematical equations [shown in the paper (“Appendix 2”)] that are used in order to do theoretical and practical calculations for power grid networks. They are used in many practical and theoretical calculations supporting finding the current and future possible states of the power grid network. The authors are convinced that in proposed approach a significant progress is made comparing to the other similar papers related to power grid network stability, when mostly only the topological conditions are considered. Here we also focused on real system workload (electrical energy passed through the network).

Another motivation for more practical approach presented in this paper is the problem of power network grid dynamics and stability in current times. It seems to be very important from different perspectives, and can be a big challenge, referring to the problems with electrical energy supply and high costs of its production due to the prices and the lack of coal, oil, gas, etc. It can be supposed that the increasing demand on electrical energy can cause different critical states of the power grid and the failures of network, for example due to weather conditions, can be a significant problem in its stability and processed task—the supply of electrical energy.

## PLANS

**Figure 2:**
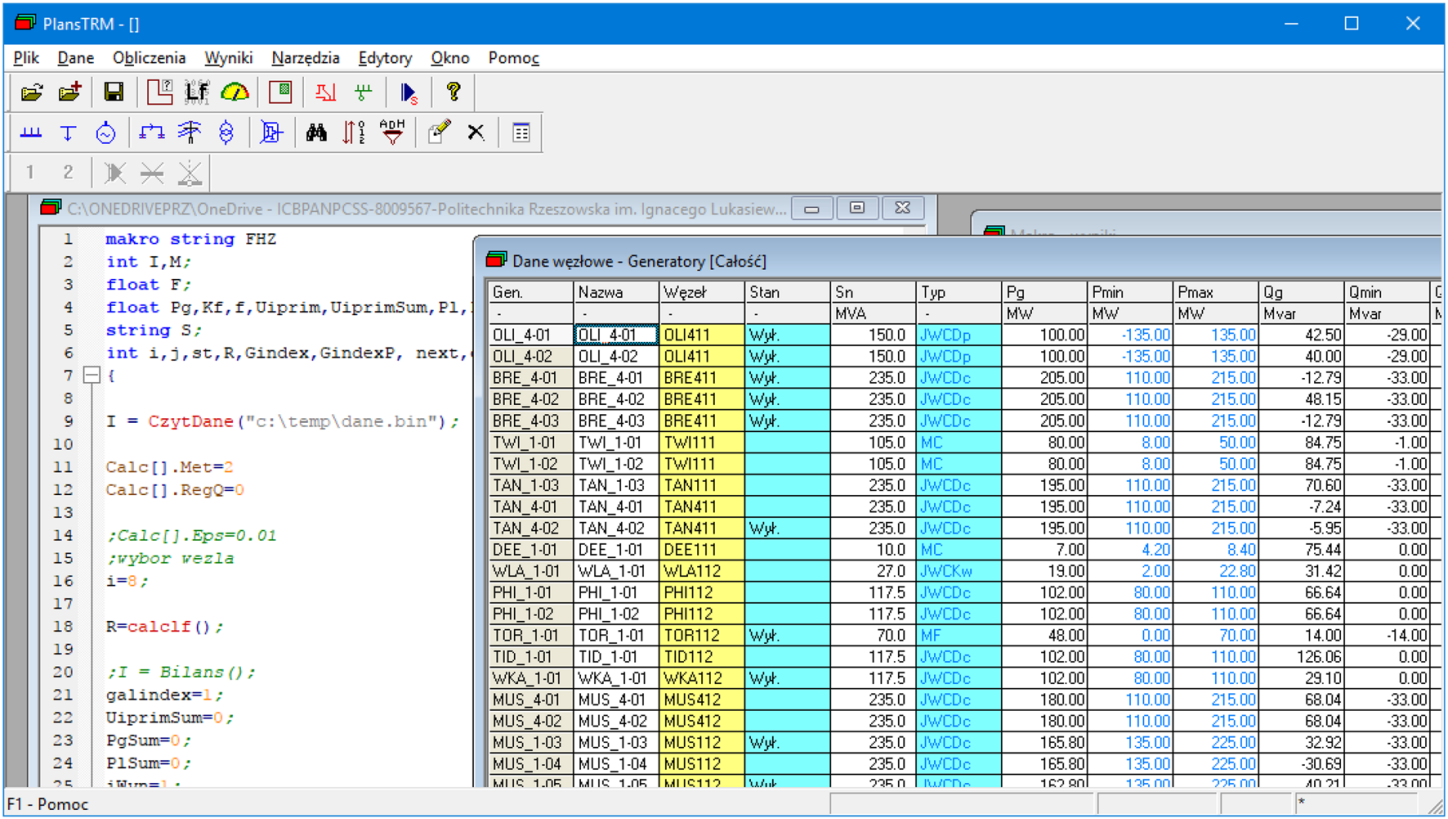
PLANS main window.

PLANS (see Fig. 2) is a Polish software^10^ widely used by many engineers for almost 20 years to simulate the load and flows in power grids. Throughout the years of its presence on the market, it has gathered a large group of Polish DSO’s: PGE, ENEA, TAURON and ENERGA^40^, for whom workshops improving competences in its use have been organized for years. Its main function—the calculation of flows in power grids—and its support for the native network data storage format have undoubtedly contributed to its popularity in Poland.

PLANS is used to make the topological analysis resulting from a graph theory approach as realistic as possible, so that the results obtained show the current behavior of the power grid with respect to real electrical parameters (voltage, current, flows, $\ldots$). Software supports data in the well-known Polish KDM format, which is used for years in the Polish power industry, as well as in the EPC format^10^ and binary format (BIN files). EPC format is a simple text file containing only power flow data. The format was developed by General Electric and used in its PSLF software^41^. BIN format is not a human-readable file, which can contain the same data as KDM or EPC, but in a compact form.

PLANS represents nodes by several types. The basic types of nodes are labeled as follows^10,42^:

- *PQ* is a load node (in such a node the active and reactive power is known, and the voltage is calculated),
- *PV* is a generation node (also called generator or power plant node—here the active power and voltage module are given, and the reactive power and voltage angle are calculated—this results from the fact, that the voltage is kept constant by the voltage regulator, and the power of generators is also known),
- *PV* with limited reactive power production (if the reactive power exceeds $Q_{max}$, the marking changes to $-1$, if $Q_{min}$ it changes to $-2$),
- the last node type is the balancing node. In practice, the balancing node is always marked with the highest number, which often indicates the total number of nodes. Inappropriate assignment of node numbers (types) may cause isolation of the subgrid in which there is no connection to the balancing node, resulting in lack of cohesion. There should always be one balancing node in the power grid—type 4; the lack of this type of node leads to a divergence of the iterative process. Usually the balancing node is the one responsible for covering the power losses in the grid, e.g. the power plant. The literature^42^ says that the phase angle of the voltage equal to zero in the balancing node should be taken.

Power grid data can be displayed in the form of tables, it is also possible to display data (for a single element) on specially prepared program windows:

- branches—data describing power grid topology and impedance parameters of lines and transformers,
- lines—parameters specific to transmission lines,
- transformers—parameters including transformer ratio and other data specific to transformers,
- nodes—data describing nodes (high voltage (HV) buses),
- generators—data describing single generators connected to the nodes (HV buses),
- loads—data describing single loads (HV/Medium Voltages (MV) transformers) connected to the grid nodes (HV buses),
- areas—division of the power grid into areas and sub-areas.

The power flow calculations are used to determine nodal voltages for preset grid loads and for preset topology and configuration. The iteration of nodal voltages can be performed after loading the data or editing power grid data—changing the connection layout, switching off elements, changing the power grid load or changing preset voltage levels in power plant nodes. With a tool like PLANS, all we have to do is write the right script to perform the analyses we need. Thanks to Macro Language for PLANS—JMP (Polish acronym for ”Jȩzyk Makropoleceń dla programu PLANS”)—a programming language that allows users of the PLANS package to automate repetitive computational activities frequently performed using this package (most details about this language are available in PLANS help system) one can design automatic actions performed with the PLANS program. For example, a program written in the JMP language can change grid parameters, perform flow calculations, select branches and nodes with overruns and save them to a file. Then it can make line exclusions, count the flow again and add the exceedances to the previous file. The user only has to write his procedures correctly in JMP and the rest will do itself. Furthermore, the same program can be used when operating on multiple data. Many program functions have been named in Polish, due to the origin and market where the software is used.

## Calculation method

The calculation of power flows aims to determine the steady state in a power grid, assuming a symmetrical load on the nodes^42,43^. The input variables are usually node powers and voltages (some). As a result of the calculation, the power flows in the power grid branches, the power losses in the system elements and the nodal voltages and powers are obtained.

The node can be characterized by 4 variables:

- active power,
- reactive power,
- node voltage modulus,
- phase angle of the voltage.

The choice of known and sought quantities is arbitrary and depends on the needs and the chosen system model. To determine 2 quantities, it is necessary to provide information about the other two. For the calculation it is necessary to determine the type of node:

- load node—received power *P* and *Q* is known,
- power plant node—known active power *P* and voltage module *U*,
- balancing node—known voltage module *U* and phase $\varphi$. It is a power plant node whose task is to balance the missing power in the nodes. In the system model 1 such node is assumed.

PLANS, based on Newton’s method (widely used in many variants, such as^44^ or^45^) was utilized in presented paper as an iterative approach, which means that the results are obtained basing on previous successive model calculations^43^. To see how this method works, please see “Appendix 2”). For each successive step in the method, some corrections—these are usually voltage corrections—are taken into account for the modulus and phase angle in the nodes. In case of power distribution equations, the following should be taken into account:

- whether the method converges,
- the computation time of a single iteration,
- the number of iterations to obtain sufficient accuracy.

**Figure 3:**
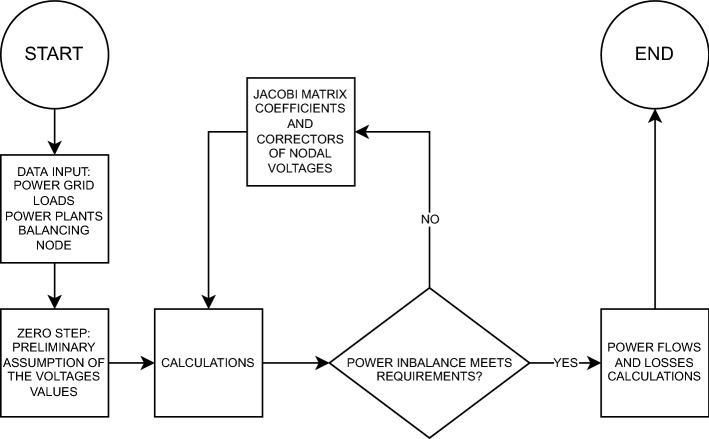
Newton method algorithm used in PLANS for analysis.

This method converges quickly (a small number of iterations is required to reach convergence), but depending on the chosen starting point can lead to a non-basic solution. The iteration process is as follows^46^ (also see Fig. 3):

1. Data input (power grid topology and parameters, *Y* matrix, node loads), nodes are marked according to type.
2. Preliminary assumption of the values of the voltages in the nodes—zero step (e.g. flat start, when the voltages in the zero step are equal to the nominal ones).
3. Based on the equations $I = Y \cdot U$ and $S_i = U_i \cdot I_i*$, the power imbalance is calculated.
4. Calculate the coefficients of the Jacobi matrix.
5. Solve the linear system of equations—obtain the corrections for angles and nodal voltage modulus and use them to correct the nodal voltage values. Go to step 3 in the next iteration.
6. The current and power flows and losses in the power grid elements are calculated and the power flow calculations are completed—the analysis of the results is carried out.

## Monte Carlo

The fundamental advantage and principle of the Monte Carlo method is the ability to obtain reasonably precise information by repeating the experiment many times. The time when Monte Carlo simulation in its current form was developed is considered to be 1949, when the article “The Monte Carlo Method” was published^47^. The formal prototype of the method can be considered to be statistical sampling, which was in use long before the publication of this work. However, the time when the method was developed is very important—it was then the first computers were created that were able to carry out such labor-intensive calculations. Without them, the possibilities of using this technique would have been drastically limited.

Since its inception, Monte Carlo simulation has been used to assess the impact of risk in many real-world scenarios, such as artificial intelligence^48^, share prices, sales forecasting^49^, project management and price determination^50^.

Furthermore—compared to forecasting models with fixed input variables—this method provides a number of advantages, such as the ability to perform sensitivity analysis or calculate correlations of input variables. Sensitivity analysis allows decision makers to see the impact of individual input variables on a given outcome, and the observed correlation helps to understand the relationship between any input variables.

**Figure 4:**
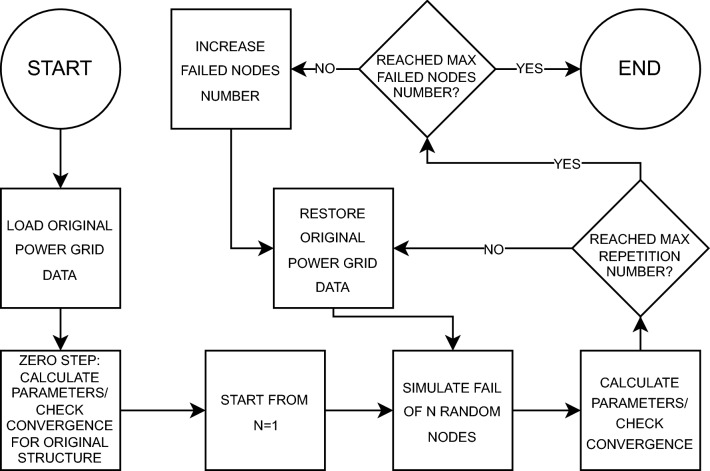
Generalised scheme of Monte-Carlo algorithm used in simulation process.

In this work, it was decided to use this method because it allows to extract averaged values from the simulation process. The application of this method consisted of performing a large number of triplicates of the node draw process for failure simulation (initially from 1 all the way up to 5), and performing calculations to analyze the state of the structure. The generalized scheme of the process conducted in this paper (both in PLANS and NetworkX) is shown in Fig. 4. After performing over 20 billion iterations in total, the averaged results were obtained, and this is presented in “Case study” section.

To briefly describe the application of Monte Carlo along with the Newton–Raphson methods, the process begins with the loading of data on the power system under study and checking data consistency. Next, the variable *N* is taken, which indicates the number of random nodes selected for simulation process. Now, using Newton–Raphson method (see Fig. 3), power flows are calculated. The consistency and correctness of the data is checked again. If the algorithm has not performed the required number of simulation calculations for a given *N*, the initial system state is restored and simulation of another *N* random nodes is performed. If the required number of repetitions has been performed, the algorithm checks whether the last *N* is equal to the assumed maximum number of failed nodes. If so, the calculation is over, if not, it increments the variable *N*, restores the system to the initial state and returns to step simulating the failure of *N* random nodes.

In this particular model (KDM in PLANS), each node has real, different parameters, so the failure of one selected node will not have similar effects as the failure of another.

## Case study

Interactive techniques have successfully guided domain experts through the complex exploration of large networks. The ever-increasing computational capabilities of computers have made it possible to process networks with many millions of vertices and analyze changing parameters in real time. This would not have been possible without many years of work by software developers to harness the available computational power for analysis. In the following section, two software packages will be used to perform analyses on complex structures: PLANS for KDM power grids and NetworkX, for its mathematical graph representation.

### Analysis in PLANS

**Figure 5:**
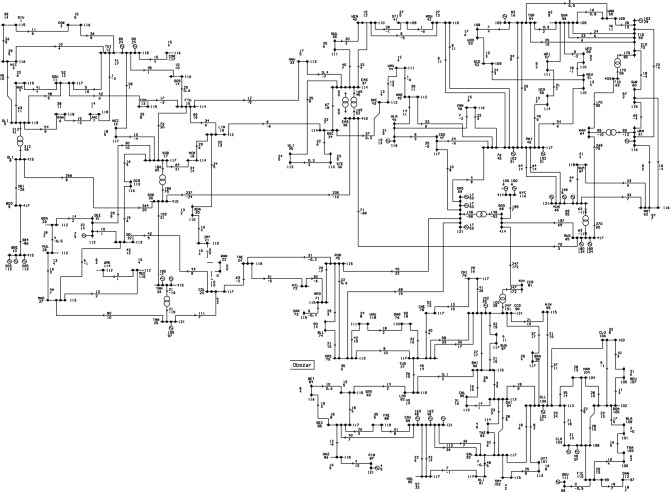
IEEE118 test model adopted to Polish conditions (KDM format in PLANS).

The authors believe that performing tests on this model with characteristics close to the real one will significantly improve the reliability of the obtained results. The second point is that analyses on real grids should use classified diagrams which, for the safety of the national power industry, should not be disclosed to the public (Authors would like to thank Z. Zdun and T. Zdun for delivering some data available in Fig. 5). We consider critical disaster—total destruction/failure of a node as the worst possible case for every tested node.

PLANS supports automation of importing binary grid data process in scripts. To begin the work with desired power grid, one needs to load its data to the program memory by calling:

$${\mathtt{I}} = {\mathtt{CzytDane}}(``{\mathtt{c}}: {{\backslash}\mathtt{data}}{{\backslash}\mathtt{grid}}.{\mathtt{bin}}'');$$

Once power grid data is loaded, there are many possibilities of manipulating nodes and transmission lines. The proposed code snippet, (see “Appendix 1”), disables the specified nodes (each node is assigned a unique number), thereby simulating its failure. The whole process is automatic and by running the prepared script code for each node *i* (randomly selected in simulation process; at a later stage also for several nodes) and then performing the most important function, which is calculating the convergence of the grid:

$${\mathtt{R}}={\mathtt{calclf}} ()$$

It is possible to determine whether the power grid is still stable or not. If the network is not able to operate with the configured topology, PLANS will return an appropriate network non-convergence message.

In our case, network calculations have been performed for the random failure of every single node, and at the same time every possible two nodes, three nodes etc. Continuing with such a procedure, aggregated results were obtained and are shown in Table 1. For the 5 failed nodes, the power grid was not able to obtain convergence state in any case.

**Table 1:** Results of the PLANS analysis.

Failed nodes	% of grid system failures
1	96.78%
2	99.23%
3	99.95%
4	99.99%
5	100%

Table 1 shows for how many cases of random removal of *n* nodes PLANS cannot calculate anything. In the PLANS program, simulating even single node’s failure quite often (more that 96%—see the Table 1) resulted in a situation that the network was not able to function at all. It comes down to the fact that the algorithm calculating the energy balance in the grid is not able to stabilize this parameter, as a result of which the physical structure of the grid would behave in the same way—it would not be able to function at all. In the case of the analyzed graph of the complex grid, the situation is different—the parameters change their values in a smooth way, gradually showing the degradation of analyzed structure properties—more details are given in “Analysis in NetworkX” section.

### Analysis in NetworkX

NetworkX is a popular Python package for creating, manipulating and studying the structure, dynamics and functions of complex networks. It has the ability to import mathematical forms of graphs from many popular formats. After loading the graph into the program, using additionally the Python language, an experienced user has wide possibilities of manipulating graph elements and performing complicated analytical processes. NetworkX is used by mathematicians, physicists, biologists, computer scientists and social scientists^51–54^.

**Figure 6:**
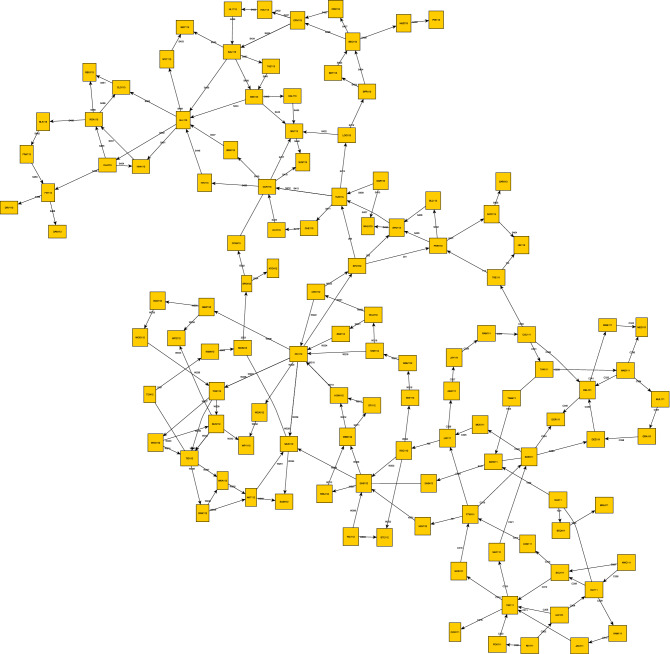
Original power grid reworked as a mathematical graph (yEd/NetworkX).

In this Section, the power grid data (based on the 220kV and 400 kV connections) obtained from modified IEEE 118 bus for Poland (data from Fig. 5 has been entered manually into the yEd software and finally imported into the NetworkX software package). The data was analyzed in the form of a mathematical graph. According to the newest report presented in December 2021^55^, Poland had 281 power transmission lines with a total length of 15,316 km. Another report^56^ has shown that this system is generally very stable and reliable if we refer to the international System Average Interruption Duration Index (SAIDI)^57^, but in Poland the DSO’s have to deal with big lengths of transmission lines and big distribution areas to balance the asymmetric production of green electricity power and, as a consequence of this situation, the problem of so-called “re-dispatch” appeared^58^.

It is visible when the grid operator forces some power stations to lower the energy production because of a region oversupply, and other plants located in a low-production region are forced to increase the output. A typical example of such a situation is a hot day in summer or a cold day in winter, when air conditioning and heating systems are used en masse respectively^59^.

In our case study, each grid node in Fig. 5 represents a separate vertex of the graph in Fig. 6. The connections between nodes were reconstructed as graph edges. This means that the network of graph connections is the same as the real KDM transmission grid model in PLANS.

The script was run in the Python language interpreter. The purpose of the code is to perform a high accuracy statistical analysis for all possible cases of node failures in the analyzed power grid. Cases were analyzed up to 5 nodes removed. The justification for this choice comes from the PLANS program, based on the results of the overall network balancing calculations—see Table 1. By the Monte Carlo method random removes of nodes in PLANS were tested and the probability that PLANS will be able to calculate the grid stability was noted. Referring to “Analysis in PLANS” section, a removal of any 5 nodes from the real power grid structure practically guarantees non-functioning (understood as the lack of Newton’s method convergence) of the considered grid system.

From the Table 2 it can be seen that despite the average global efficiency is still relatively high (change from the original value of 0.211691 to 0.202462—a decrease of only 4%), although the results in the PLANS do not reflect this. Table 2 suggests that on average the situation is not much worse than it was but taking into account the values of the minimum local efficiency this parameter changes in a significant way—a decrease in the value by 40%. In the case of this parameter, we considered a worst possible scenario for the whole simulation process as an analogy to the simulation process in PLANS, where the lack of network balance makes it completely impossible to operate. In the case of power grids, the local efficiency parameter seems to be more important considering the worst possible situation^4^.

**Table 2:** Graph simulation results (NetworkX).

Failed nodes parameters	Global efficiency	Minimum global efficiency	Average clustering coefficient	Minimum local efficiency
0 (original)	0.211691	0.211691	0.168032	0.175659
1	0.209994	0.192695	0.166500	0.153961
2	0.208472	0.178102	0.164964	0.133100
3	0.206406	0.164621	0.163118	0.124692
4	0.204678	0.153897	0.161576	0.112336
5	0.202462	0.131334	0.160204	0.102921

**Figure 7:**
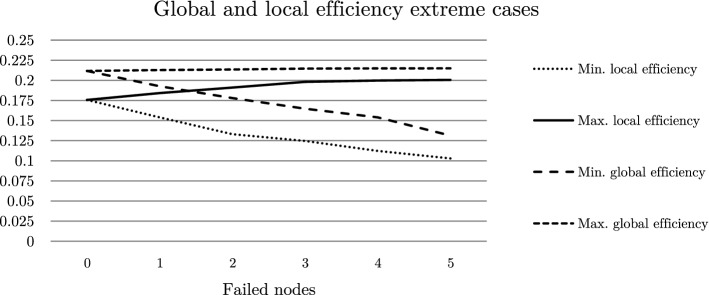
Extreme cases global and local efficiency values during simulation.

Considering Fig. 7 an increasing discrepancy between the extreme values of the local efficiency parameter can be seen. This is in contrast to the global efficiency parameter; its average value after all stages of the simulation did not differ significantly from the initial value. The average value can only be considered to a limited extent because of its insignificant fluctuations. On the other hand, considering its minimum value as the worst possible situation opens an analogy to the analysis performed in the PLANS program.

Table 2 with NetworkX data shows, that the graph, which is a representation of the power grid model, does not react as drastically in the simulation process as the original model, taking into account the obtained parameter values.

It is worth to note here especially the obtained values of the local efficiency parameter. In the extreme case for 5 failing nodes, this value drops by more than 40% (particularly noticeable in Fig. 7). This cannot be compared with the previously mentioned behavior of the model in PLANS.

**Figure 8:**
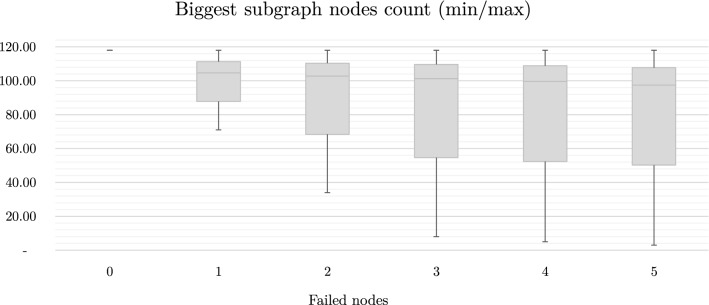
Extreme values of the biggest subgraph nodes number.

For the results obtained in the graph simulation, one can see how much the transmission structure becomes fragmented, when 3 or more failing nodes are considered. This can be clearly seen in Fig. 8. While such fragmentation does not necessarily affect typical graph parameters, in the case of an electrical network it becomes an obstacle to any form of operation. In the case of a mathematical graph, if one or more nodes (vertices) are removed from the structure, the graph may be divided into several smaller subgraphs. In an analogous situation for the power grid, it is certain that if parts of the transmission system are completely separated, its operation is highly questionable. Moreover, it is clear from the simulations performed that only in a negligible percentage of cases the system continues to maintain its operation.

## Summary

From a topology point of view, the analyzed power grid looks to be in a good shape, but considering only the worst case, it can show how the electrical network is topologically vulnerable to node failures. As can be inferred from the results, this particular real power grid is not very fault-tolerant, so it may not look to be the best for analysis.

In the case of simulations performed on a graph, not all parameters have changed significantly their values during the simulation process. This draws particular attention to how useful specialized software dedicated to specific, narrow fields of application is. The main reason for this are the unique parameters. In the case under consideration, where we are dealing with a power grid, these will be the parameters describing the real electrical characteristics of the nodes and transmission lines. While in the case of performed in PLANS simulation the grid was extremely sensitive to topological changes (failures) in the model structure, in the analysis of the mathematical graph the sensitivity of the model is incomparably less. Such a difference in behavior confirms the fact that simulations performed in specialized software undoubtedly give a whole more complete insight into the internal behavior of the network. They are able to reflect the characteristic parameters which in no way can be be represented in a simple complex network graph.

According to the best authors’ knowledge this is the first attempt to use the Monte-Carlo method to compare failure simulations performed both in specialized power grids flows software and in a classical mathematical graph representation. The future research related to this topic can be focused on the problem with random edge removal, where the failures will have in some cases different nature. Also as an extension of current work, one can try to extend both structures and add redundant connections to act as backup bypasses. While this is not a big problem in the mathematical graph, the addition of power lines (connections) in the PLANS must be deliberated and specific, with analyses of short-circuit spreads and currents necessary before interconnection. Usually it turns out that the $S_k$ power (short-circuit power) changes so much that adjustments to protection settings will be necessary at each node. In its present form, the model is optimally configured and tuned. Such an analysis goes far beyond the scope of this paper. Further, the creation of extended scenarios related to failure, attacks or deletion of nodes may be considered, including the use of a completely larger data model that includes the power grid structure of European countries.

One can use tools to analyze complex electrical power grid networks, but should be aware that the worst case scenarios should be taken. Generalization of such a situation is somewhat limited, but in this particular case it is true. Taking into account article^36^, topological graph parameters do not look too bad, but in the case of electro-network graph any network operation is mostly impossible 1. In the case of^4^, we have over 5 times more analyzed elements, and extending their findings we can conclude that the most valuable results of such parameters analysis is the worst case analysis—and this has been proven by PLANS simulations with Monte Carlo approach.

## Data Availability

The datasets generated and/or analysed during the current study are not publicly available because of the the national power industry safety but are available from the corresponding author on a reasonable request.

## Appendix 1: PLANS simulation code

The code for simulation in PLANS:

## Appendix 2: Newton–Raphson method algorithm

The details related to the Newton calculation method.

Taking the number of load nodes as *L* and power plant nodes as *G*, the number of nodes in the model can be defined as:

4 $$\begin{aligned} L + G + 1 = N. \end{aligned}$$

Therefore, the state vector *X* has $N-1$ coordinates that represent the voltages at the nodes (balancing node voltage). The voltage can be represented in trigonometric and algebraic form:

5 $$\begin{aligned} {\underline{U}}_i=U_ie^{j\delta _i}={U_1}_i+j{U_2}_i, \end{aligned}$$

where $\delta$ is the voltage phase and $i=1,2,$..., $N-1$.

The coordinates of the state vector can be $N-1$ modules of *U*, and $N-1$ phase angles $\delta$, but the vector can also represent the components $U_1$ and $U_2$. Other quantities, such as powers, power losses and branch currents, are determined from the differences in node voltages. The state vector has the form: $$X^{T} = \begin{bmatrix} U^{T} &{} \delta ^{T} \\ \end{bmatrix}$$ or $$X^{T} = \begin{bmatrix} {U1}^{T} &{} {U2}^{T} \\ \end{bmatrix}$$.

Power nodes (power flowing into the node) and node voltages are related by a relationship:

6 $$\begin{aligned} {\underline{S}}_i={\underline{U}}_i{{\underline{I}}_i}^*={{\underline{U}}_i}^2{{\underline{Y}}_{ii}}^*+{\underline{U}}_i\sum _{j=1,j\ne i}^{N}{{{\underline{U}}_j}^*{{\underline{Y}}_{ij}}^*}. \end{aligned}$$

The voltages are assumed to be interphase, so that the calculated power is three-phase. The nodal and load powers must be equal:

7 $$\begin{aligned} S(X) - S_{Z} = F(X) = 0, \end{aligned}$$

where *S*(*X*) is the vector of calculated nodal powers and $S_Z$ is the set power. This gives the equation $F(X)=0$. Using the state vector, where the coordinates are described by the modulus and the phase angle, the equations can be described as:

8 $$\begin{aligned}&{{\underline{U}}_i}^2G_{ij}+\sum _{j=1,j\ne i}^{N}{U_iU_j\left[ G_{ij}\cos {\left( \delta _i-\delta _j\right) }+B_{ij}\sin {\left( \delta _i-\delta _j\right) }\right] -P_i}=0, \end{aligned}$$

9 $$\begin{aligned}&-{{\underline{U}}_i}^2B_{ij}+\sum _{j=1,j\ne i}^{N}{U_iU_j\left[ G_{ij}\sin {\left( \delta _i-\delta _j\right) }-B_{ij}\cos (\delta _i-\delta _j)\right] -Q_i}=0\ . \end{aligned}$$

As a result of Newton method work, $N-1+L$ equations are obtained. At the same time, the voltages at the power plant nodes (in *G* number) are known. This means that the number of equations and the set voltages are the same:

10 $$\begin{aligned} (N-1+L)+G=(N+L+G+1)-2=2(N-1). \end{aligned}$$

In some situations Eq. (10) can still hold and be correct under certain conditions for remaining nodes that have not failed. But the overall summary results of simulation are not convergent because the disconnected part of the system is not supplied by the appropriate amount of electrical power (it was for example completely disconnected from the power plant nodes) or the power plant nodes haven’t got the required amount of electrical power necessary for normal operation.

In the Eqs. (8) and (9), quantities can be distinguished:

11 $$\begin{aligned} {\underline{S}}_i=P_i+jQ_i, \end{aligned}$$

that is, the nodal power of the *i*-th node, and:

12 $$\begin{aligned} Y_{ij} = G_{ij} + jB_{ij}, \end{aligned}$$

which represents the grid admittance matrix elements.

The presented equations refer to mathematical model of the grid in steady state. The following element models can be distinguished:

- Power grid—*Y* matrix,
- Load—*P* and *Q* capacities,
- Power plants—*P* power and *U* voltage,
- Balancing node—*U* voltage and its $\delta$ phase.

The system of equations determined in this way is solved using iterative methods^60^. One of them is the Newton–Raphson method. It uses linear approximations of the function increments (decomposition into Taylor series):

13 $$\begin{aligned} {F}\left( {X} \right) \approx {F}\left( {X}^{k} \right) + {J}^{k}{\Delta }{X}^{k}, \end{aligned}$$

where *J* is the Jacobi matrix. The correction sought is:

14 $$\begin{aligned} {\Delta }{X}^{k} = - \left( {J}^{k} \right) ^{{- 1}}{F}\left( {X}^{k} \right) . \end{aligned}$$

The iterative dependence is given as:

15 $$\begin{aligned} {X}^{k + 1}{=}{X}^{k}{+}{\Delta }{X}^{k} = {X}^{k} - \left( {J}^{k} \right) ^{{- 1}}{F}\left( {X}^{k} \right) . \end{aligned}$$
